# Ureterocele urothelial carcinoma: managing a rare presentation

**DOI:** 10.3332/ecancer.2016.621

**Published:** 2016-02-16

**Authors:** Juan Carlos Astigueta, Milagros Abad-Licham, Eloy Silva, Víctor Alvarez, Francis Piccone, Enrique Cruz, Joan Palou Redorta

**Affiliations:** 1 Servicio de Urología Oncológica, Instituto Regional de Enfermedades Neoplásicas, Trujillo, Perú; 2 Servicio de Patología Oncológica, Instituto Regional de Enfermedades Neoplásicas, Trujillo, Perú; 3 Servicio de Radiología, Instituto Regional de Enfermedades Neoplásicas, Trujillo, Perú; 4 Facultad de Medicina, Universidad Privada Antenor Orrego, Trujillo, Perú; 5 Servicio de Urología, Fundacio Puigvert, Barcelona, Spain

**Keywords:** urothelial carcinoma, ureterocele

## Abstract

It is very uncommon for urothelial carcinoma to develop in an ureterocele. It is generally discovered in an imaging study or in connection with haematuria.

We found very few reports in the literature. Here, we report on the case of a 71-year-old male who initially presented with haematuria and low back pain and who then underwent transurethral resection for an intraureterocele tumour. Pathology confirmed urothelial carcinoma.

## Introduction

Urothelial carcinoma is the second most common form of urologic oncology pathology and is most often found in the bladder (90%) followed by the renal pelvis, ureter, urethra, and embrionic remnants [1, 2].

The ureterocele is a pseudocystic dilatation of the terminal segment of the ureter. Its aetiology is unknown. It usually occurs in a double collection system and is diagnosed as part of an examination of secondary symptoms related to obstruction problems [1, 3, 4].

There are few reported cases of malignant tumours of the ureterocele, specifically urothelial carcinomas. To date, there is no standardised management protocol [5–16]. Here, we report on such a case, as well as data from the literature review, mainly focused on a diagnostic and therapeutic approach.

## Case report

**Figure 1.**
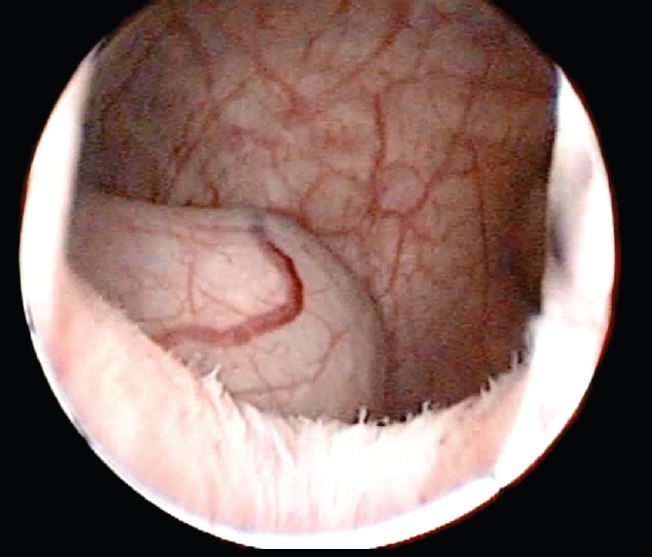
Urethrocystoscopy: bladder, note a rounded, thin-walled, translucent tumour consistent withaureterocele.

**Figure 2.**
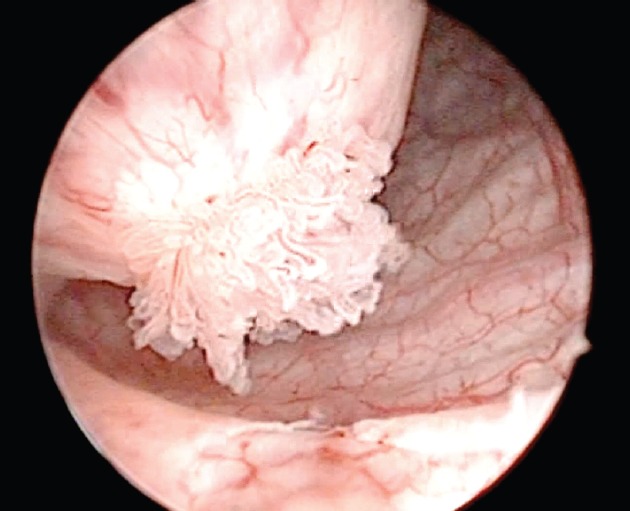
Urethrocystoscopy: note the multiple papillary lesions on the medial face visible through the hole leading to the right ureteral meatus.

A 71-year-old male patient presented with a two-month-old illness characterised by intermittent gross haematuria and right lower back pain. An ultrasound study revealed a hypoechoic image in the bladder that was round with regular borders, 35 × 33 mm in diameter. The physical examination was not remarkable. Cystoscopy identified, in the right haemitrigone area, a 4 × 3 cm rounded tumour with thin translucent walls consistent with a ureterocele (Figure 1). On its medial face, through a hole leading to the ureteral orifice, a papillary lesion (0.5 × 0.3 cm) protrudes and seems to originate within the ureterocele. The left urethral orifice is well established and excretes clear urine. No other lesions were observed elsewhere in the bladder (Figure 2).

**Figure 3.**
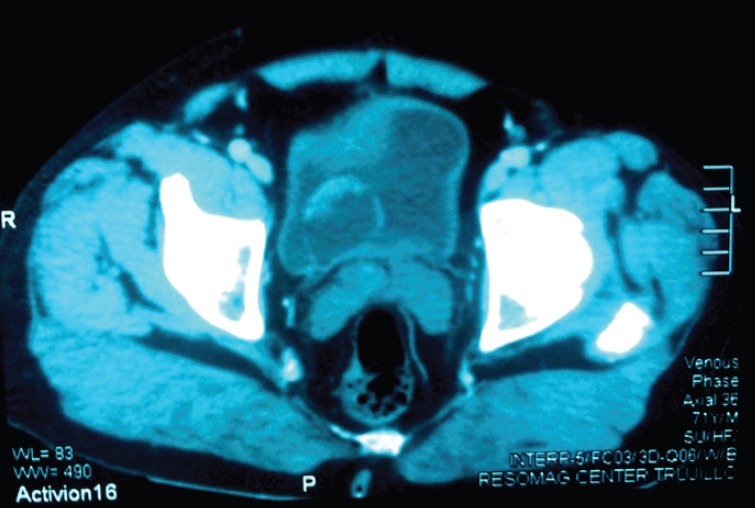
Scan: observe at the intravesical level, a hypodense saccular image (2.5 × 4 cm) with irregular mural thickening at the anterior-superior edge.

Visible in the scan at the intravesical level is a hypodense saccular image (2.5 × 4 cm) with an irregular 10 mm mural thickening at the anterior-superior edge displaying homogenous enhancement after contrast administration. Other organs fall within normal parameters (Figure 3).

**Figure 4.**
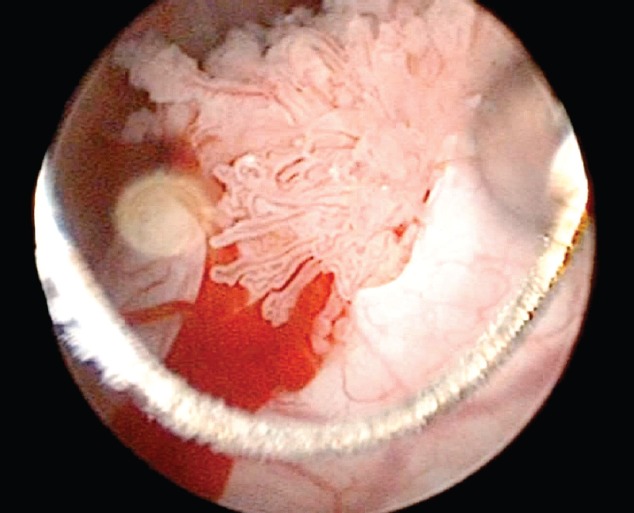
Transurethral resection: upon removing the ureterocele dome, one can observe a papilary lesion whose base is attached to the inner surface of the same.

A transurethral resection (TUR) was performed and confirmed the cystoscopic findings. Upon removing the ureterocele dome, one can identify a papillary lesion whose base is attached to the inner surface of the same. The proximal portion of the ureterocele is free of gross disease, but the medial face appears swollen. It was resected by separating the tumour adhering to the ureterocele walls and base (Figure 4). Once the resection was complete, a dilated ureter free of malignant-appearing lesions is visible.

**Figure 5.**
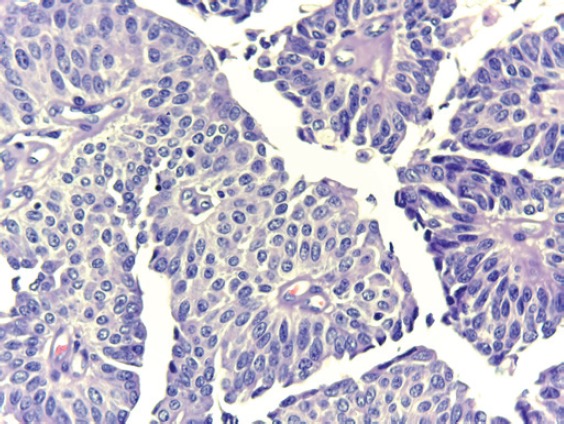
(40X) H&E: Microphotograph at higher magnification showing a cell disorder, mild nuclear atypia, and fibrovascular cores.

In the laboratory, we received multiple fragments of tissue for study, making up a total volume of 10 cc, brown in colour and elastic. Histologicial diagnosis indicated low-grade urothelial papillary carcinoma, with a suburothelial and muscle layer free of neoplasia. Figure 5 shows the microphotograph.

The patient improved in the final control test, nearly a year after surgery. We see no ultrasound images with lesions compatible with the recurrence or progression of the disease. Urine cytology was negative for malignancy, and endoscopically, we observed a flattened scarred area on the periphery of the right urethral orifice; no lesions suggestive of malignancy were found. In the dilated ureter, there were no suspicious lesions. Regarding renal function, serum studies indicated normal levels of creatinine and normal creatinine clearance. No hydronephrosis or other abnormality was observed using ultrasound. To date, there are periodic checks of urine cytology, imaging, as well as endoscopic studies.

## Discussion

Ureteroceles are pseudocystic dilations of the terminal segment of the ureter that protrude into the bladder. Their walls are made up of a thick muscle layer and collagen interposed between the urothelium bladder and the ureter [1]. Their size may range from a few milimetres to several centimetres and they are associated with anomalies such as vesicoureteral reflux, utereopelvic duplication, and renal dysplasia [4].

The specific mechanisms behind the origin of ureteroceles are still not elucidated. The theories that seek to explain their pathogenesis fall into two categories: (1) those that propose a congenital role (incomplete canalisation of the Chwalla membrane, intrinsic deficiency of the muscular component of the distal ureter, etc. and (2) those that suggest a secondary origin due to a narrowed ureteral orifice resulting from inflammatory infections processes that produce fibrosis and stenosis of the opening and promote development of the ureterocele [1, 4].

The presence of tumours in a ureterocele is extremely rare and only a few cases have been documented; these includepheochromocytoma (Cabanas *et al*. 1973) [5], leiomyoma (Sekar *et al*. 1980) [6], adenocarcinoma (Yenilmez *et al*., 2007) [7], and urothelial carcinoma [8–16]. The development of urothelial carcinoma is due to the presence of urothelial tissue coating the same and preserving its capacity to become malignant [15].

Imaging studies play a key role in the initial diagnosis of ureteroceles or intraureterocele lesions. In an ultrasound, a rounded cystic intravesical thin-walled structure usually appears located at the base of the bladder [4]. Furthermore, as described by Andrew *et al*., there are features present such as irregular echogenicity and the absence of acoustic shadowing that permit a differential diagnosis and suggest the presence of an intraureterocele tumour [10].

Excretory urography is another important test that allows one to identify an intravesical defect that is radiolucent and globular, described as ‘indicating a cobra head’, which refers to the expansion of the distal ureter surrounded by a thin radiolucent line observed in patients with adult-type ureterocele (orthotopic) [17–19]. These changes are also tomographically evaluated in this report and permit one to better define the wall and content images – they also provide information on extravesicaldisease [19]. Any thickening or irregularity of the ‘hood’ of the cobra must be described as a pseudoureterocele. The same can result from oedema associated with a stone or tumour of the distal ureter or the ureter orifice, simulating a ureterocele in the urography, as described by Morse and Ochi [20–21]. If the appearance is not ‘classic’, cystoscopic evaluation must be conducted as soon as possible.

In the case presented here, the patient is male and the ureterocele was right unilateral, unlike those described in most reports; no associated abnormalities were found. The probable diagnosis was determined from the imaging studies, as noted earlier, performed as part of the evaluation protocol for haematuria – the most common sign as per the reviewed literature. Urethrocystoscopy confirmed the diagnosis and enabled the choice of an initial therapeutic approach based on transurethral resection of the tumour, as described by the majority of similar case reports [11, 12, 14–16]. We did a complete resection to perform an in-depth separation of the ureterocele from the tumour and the perimeatal base. No re-TUR was called for given the low-risk muscular-negative histology and that these factors are the most persuasive indicators in favour of a second resection [22].

A definitive treatment is suggested by histological results and may involve radical surgery as in the cases presented by Heyman and Garcia [9, 15], more conservative resection of the ureterocele with a segment of the terminal ureter and re-implantation [10–12], or as in our case, simply observing and monitoring.

The histological diagnosis of transitional cell carcinoma (TCC) or urothelial carcinoma (WHO 2004) [2] of the ureterocele, as in this case, very rarely turns up in the PubMed, EBSCO, and BVS databases. Only nine (09) case reports were found, of which six were from the last century and of the remaining three, the most recent was a decade ago. Due to the age of the reports, the the two reviewed databases only showed titles and abstracts. According to our review, Perego *et al*. in 1974 published the first case report on this entity, reporting that at that time there were no clinical, radiological or endoscopic elements that allowed the differentiation of it from a simple orthotopic ureterocele [8]. Heyman *et al*., ten years later, in 1984, reported on another ureterocele case with cancer developing in a remaining distal ureter. In Japan, according to Ishida *et al*. (2002) [14], there have been eight reported cases; however, Kadono *et al*., refer to only four cases since 2004 [16]. In Table 1, we show an overall summary of the data culled from the literature and we include the dates for cases shown.

The histology of low-grade urothelial carcinoma with no suburothelial or muscular compromise, coupled with the absence of disease in extension studies (ureteroscopy and urotomograph) allows us to propose TUR observation and monitoring with endoscopy, urine cytology, and imaging studies as an option for further management.

## Conclusions

The development of urothelial carcinoma in ureterocelesis very rare. Its potential diagnosis is suggested by imaging studies that reveal a thickening of the ureterocele walls. The most common sign is haematuria.

The management protocol is not defined. Transurethral resection is an alternative for initial management that enables the gathering of histological information and decisions to be made regarding a definitive treatment.

## Figures and Tables

**Video 1.**
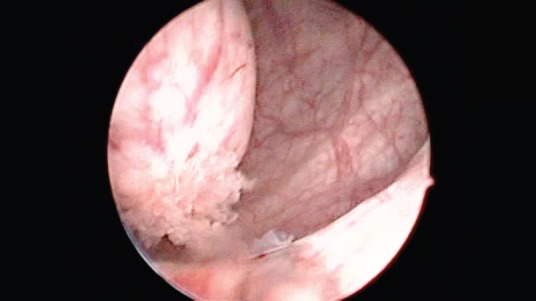
Transurethral resection of ureterocele urothelial carcinoma. To view this video, click here http://ecancer.org/journal/10/full/621- ureterocele-urothelial-carcinoma-managing-a-rare-presentation.php.

**Table 1.** List of publications on urothelial carcinoma and ureteroceles.

Author (year)/article	Age	Sex	Initial symptoms	IVU/P	US	CAT	Side	Cystoscopy	Treatment	AP
Perego *et al* (1974)/E	68 y	M	Haematuria	Compatible with UC.	No	No	D	Compatible with UC	RUU	TCC
Heyman *et al* (1984)/E	54 y	M	Haematuria	Yes	Solid mass intraUC	No	L	Compatible with UC	HNU	TCC IM
Andrew *et al* (1985)/E	–	M	–	DRP	IntraUC USs	No	R	–	URR	TCC
Nakajima *et al* (1986)/R	35 y	M	Terminal dysuria	Cobra head DRP	–	No	L	Compatible with UC	TUR, URR	TCC
Forer *et al* (1990)/E	62 y	M	Haematuria	Compatible with UC	Complex cystic mass	No EVD	L	Compatible with UC	TUR, URR	TCC
Fukunaga *et al* (1993)/T	–	F	–	–	–	–	–	–	–	TCC
Ishida *et al* (2002)/R	45 y	F	Haematuria	Compatible with UC	Compatible with UC	–	L	Compatible with UC	TUR	TCC
Garcia *et al* (2002)/E	74 y	M	Haematuria	Compatible with UC	DRP	Filling defect in UC	L	Compatible with UC	TUR CP, NU	UC IM
Kadono *et al* (2004)/E	62 y	M	Haematuria	Cobra head	–	–	L	IntraUC images	TUR	TCC
Astigueta *et al* (2015)	71 y	M	Haematuria, low back pain	–	Compatible with UC	Solid content in UC	R	Intra UC content	TUR	UC

IVU: intravenous urography; P: pyelography; US: ultrasound; CAT: tomography; AP anatomy pathology; R: review; E: extensive; T: title; R: right; L: left, M: male; F: female; DRP: dilatation of the renal pelvis; UC: ureterocele; EVD: extravesical illness; T: title; HNU: heminephroureterectomy; URR: ureterocele resection and reimplantation; TUR: transurethral resection; CP: cystoprostatectomy; NU: nephroureterectomy; TCC: transitional cell carcinoma; UC: urothelial carcinoma; IM: invasive muscle
